# Alleviation of Hepatic Ischemia Reperfusion Injury by Oleanolic Acid Pretreating via Reducing HMGB1 Release and Inhibiting Apoptosis and Autophagy

**DOI:** 10.1155/2019/3240713

**Published:** 2019-06-18

**Authors:** Wenwen Wang, Liwei Wu, Jingjing Li, Jie Ji, Kan Chen, Qiang Yu, Sainan Li, Jiao Feng, Tong Liu, Jie Zhang, Jiaojiao Chen, Yuting Zhou, Yuqing Mao, Fan Wang, Weiqi Dai, Xiaoming Fan, Chuanyong Guo, Jianye Wu

**Affiliations:** ^1^Department of Gastroenterology, Putuo People's Hospital, Tongji University School of Medicine, Shanghai 200060, China; ^2^Department of Gastroenterology, Shanghai Tenth People's Hospital, Tongji University School of Medicine, Shanghai 200072, China; ^3^Shanghai Tenth Hospital, School of Clinical Medicine of Nanjing Medical University, Shanghai 200072, China; ^4^Department of Gerontology, Shanghai General Hospital, Shanghai Jiaotong University School of Medicine, Shanghai 200080, China; ^5^Department of Oncology, Shanghai General Hospital, Shanghai Jiaotong University School of Medicine, Shanghai 200080, China; ^6^Department of Gastroenterology, Zhongshan Hospital of Fudan University and Shanghai Institute of Liver Diseases, Zhongshan Hospital of Fudan University, Shanghai 200032, China; ^7^Department of Gastroenterology, Jinshan Hospital of Fudan University, Jinshan, Shanghai 201508, China

## Abstract

Hepatic ischemia reperfusion (IR) injury (IRI) occurs during liver transplantation, hepatectomy, and hemorrhagic shock. Oleanolic acid (OA) is a natural compound with antioxidant and anti-inflammatory activity that has been used to treat liver disorders in clinical practice for several years. Here, we investigated the effects and underlying mechanisms of OA in hepatic IRI. A 60-minute partial (70%) hepatic, warm, ischemic reperfusion model was established in BALB/c mice, and two doses (30 and 60 mg/kg) of OA were administered intragastrically for 7 consecutive days prior to hepatic IR. Orbital blood and liver specimens were collected at 2, 8, and 24 h after IR. The results showed that OA preconditioning significantly alleviated hepatic injury, as evidenced by decreased alanine aminotransferase and aspartate aminotransferase levels; improved histology, inhibition of JNK phosphorylation, and high mobility group box 1 (HMGB1); and tumor necrosis factor-*α* downregulation in hepatic IR mice. OA upregulated Bcl-2 and downregulated caspase-3, caspase-9, Bax, Beclin 1, and LC3, which play crucial roles in the regulation of apoptosis and autophagy. These findings highlighted the protective effects of OA against hepatic IRI mediated by the inhibition of apoptosis and autophagy and the release of HMGB1, which acted as a late inflammatory mediator in hepatic IRI.

## 1. Introduction

Hepatic ischemia-reperfusion injury (IRI) is an important cause of liver dysfunction and a serious complication of hepatic surgery and liver transplantation. Hepatic IR elicits an acute inflammatory response, leading to the formation of reactive oxygen species and the release of inflammatory cytokines, which lead to hepatocellular damage and organ failure [1, 2]. In addition to necrosis [3], other modes of cell death such as apoptosis [4, 5] and autophagy [6–8] play important roles in the mechanisms of hepatic IR.

Oleanolic acid (3b-hydroxyolean-12-en-28-oic acid, (OA)), a natural pentacyclic triterpenoid compound that is commonly found in food and in medicinal plants in the form of free acid or triterpenoid glycosides is widely distributed in plantae around the world [9, 10]. In China, OA is used as an over-the-counter oral remedy for the treatment of liver disorders such as viral hepatitis [9]. Studies show that OA alleviates inflammation and attenuates liver injury in chemical-induced acute hepatic injury and in chronic liver fibrosis and cirrhosis in animal models, as determined by decreased liver enzymes and mitigation of hepatocellular necrosis [11–14]. OA pretreatment has protective effects on IRI of the heart and kidney during the acute phase [15–17].

The high-mobility group box 1 (HMGB1) protein is a nuclear factor and a late mediator of inflammation in sepsis [18, 19]. HMGB1 levels increase as early as 1 h after hepatic IR, and inhibition of HMGB1 activity attenuates liver tissue damage and downregulates proinflammatory cytokine expression, indicating that blocking HMGB1 may be a therapeutic target in hepatic IRI [20, 21]. *In vivo* and *in vitro* studies show that toll-like receptor 4 (TLR4) acts as a receptor for HMGB1, and the interaction between HMGB1 and TLR4 plays a key role in the mechanism of hepatic IRI [21, 22].

The aim of the present study was to examine the hepatoprotective effects of OA on hepatic IRI and explore the underlying mechanism to identify potential novel targets for the prophylaxis and treatment of liver IRI.

## 2. Materials and Methods

### 2.1. Chemicals and Reagents

OA was obtained from Sigma-Aldrich (St. Louis, MO, USA). Sodium carboxymethylcellulose (CMC-Na) was provided by Sinopharm (Shanghai, China). Alanine aminotransferase (ALT) and aspartate aminotransferase (AST) microplate test kits were obtained from Nanjing Jiancheng Bioengineering Institute (Jiancheng Biotech, China). TNF-*α* enzyme-linked immunosorbent assay (ELISA) kits were acquired from eBioscience (San Diego, CA, USA). The RNA polymerase chain reaction (PCR) kit was purchased from Takara Biotechnology (Dalian, China). The antibodies used in this study included those against HMGB1, TLR4 (Epitomics, Burlingame, CA, USA), TNF-*α*, caspase-3, caspase-9, Bcl-2, Bax, Beclin 1, LC3, c-Jun NH2-terminal kinase (JNK), phospho-JNK (p-JNK), and *β*-actin (Proteintech, Chicago, IL, USA).

### 2.2. Animals

All experiments carried out on mice conformed to the National Institutes of Health Guidelines and were approved by the Animal Care and Use Committee of Shanghai Tongji University. Male Balb/c mice weighing 21–25 g (6–8 weeks old) were supplied by Shanghai SLAC Laboratory Animal Co. Ltd. (Shanghai, China). The mice were housed in plastic cages in a temperature-controlled environment of 22°C under an alternating 12 h : 12 h light-dark circadian rhythm and were provided food and water ad libitum.

### 2.3. Experimental Design

An OA suspension was prepared with 0.5% CMC-Na aqueous solution [23]. Mice were randomly divided into five groups. Each group received an equal volume of the indicated liquid intragastrically once a day for 7 consecutive days before undergoing surgery as follows [24]:

1. Sham group (*n* = 18): mice received physiological saline followed by sham operation
2. CMC group (*n* = 18): mice received 0.5% CMC-Na aqueous solution followed by IR procedure
3. IR group (*n* = 18): mice received physiological saline followed by IR procedure
4. L group (*n* = 18): mice received 30 mg/kg OA suspension followed by IR operation
5. H group (n = 18): mice received 60 mg/kg OA suspension followed by IR operation

### 2.4. Establishment of the IR Model

A well-established mouse model of segmental (70%) hepatic warm ischemia was used [25, 26]. Mice were fasted for 24 h before the operation and then anesthetized with one dose (1.25%) of sodium pentobarbital (Nembutal, St. Louis, MO, USA) intraperitoneally. Midline laparotomy was performed to expose the liver hilum. All the structures in the portal canal (hepatic artery, portal vein, and bile duct) to the left and median liver lobes were occluded with a microvascular clamp, yielding 70% hepatic ischemia. The abdomen was covered with saline-soaked gauze during the ischemic period. After 60 min, the clamp was removed to achieve reperfusion, and the abdominal incision was sewn with 4-0 silk sutures. The sham group underwent the aforementioned procedure without the vascular occlusion. An animal body temperature maintenance instrument (ZS Dichuang, Beijing, China) was used to maintain a constant body temperature during the procedure.

### 2.5. Sample Collection

Each group was divided into three subgroups according to sample collecting time points (2, 8, and 24 h after IR operation). At each time point, six mice per group were randomly sacrificed, and the left and median liver lobes along with orbital blood were collected.

### 2.6. Biochemical Analysis

After storing blood samples at 4°C for 4–5 h, serum was obtained by centrifuging at 3,000 rpm for 10 min. Serum levels of alanine transaminase (ALT) and aspartate aminotransferase (AST) were measured using an automated chemical analyzer (Olympus AU1000, Tokyo, Japan). Serum levels of TNF-*α* were determined with ELISA kits according to the manufacturer's protocol.

### 2.7. Hepatic Histological Examination

Liver specimens removed from the left lobe were fixed in 4% paraformaldehyde and embedded in paraffin. Samples were sliced into 5 *μ*m thick sections and stained with hematoxylin and eosin (H&E). Inflammation and tissue damage were evaluated with a light microscope. Five fields (200x magnification) per slice were examined by an experienced pathologist [27, 28]. Histopathological sections were evaluated according to Suzuki's histologic grading [29]. The percentage of necrotic and edematous areas in H&E-stained liver sections was calculated using Image-Pro Plus 6.0. According to the degree of cytoplasmic vacuolization, sinusoidal congestion, and parenchymal necrosis, the liver sections were scored from 0 to 4 as previously described [28]: 0—none; 1—mild; 2—moderate; 3—marked; and 4—severe to diffuse.

### 2.8. Immunohistochemistry (IHC)

Paraffin-embedded liver sections were dewaxed in xylene and dehydrated in an alcohol gradient. Antigen retrieval was achieved using citrate buffer and incubation in in a 95°C water bath for 20 min. The sections were incubated with 3% hydrogen peroxide for 10 min to block endogenous peroxidases. Sections were washed with phosphate-buffered saline (PBS) three times, and nonspecific proteins were blocked using 5% bovine serum albumin for 20 min followed by a 10-min incubation at room temperature. Then, the liver specimens were incubated overnight at 4°C with the following primary antibodies and dilutions: HMGB1 (1 : 350), TLR4 (1 : 50), TNF-*α* (1 : 100), and p-JNK (1 : 100), followed by incubation in secondary antibody (1 : 50) for 1 h at 37°C. A diaminobenzidine kit was used to analyze antibody binding under a light microscope. The ratios of stained to total areas were measured using Image-Pro Plus software (version 6.0).

### 2.9. SYBR Green Real-Time Polymerase Chain Reaction (PCR)

Total RNA was extracted from frozen liver specimens using the TRIzol reagent (Tiangen Biotech, China). RNA was reverse-transcribed into cDNA with a reverse-transcription kit (TaKaRa Biotechnology, China). Gene expression was measured using SYBR Premix Ex Taq (TaKaRa Biotechnology, China), and the resulting cDNA was quantified with the 7900HT fast real-time PCR system (Applied Biosystems, CA, USA). Oligonucleotide primer sequences are listed in Table 1. The relative expression levels were analyzed using the 2^−△△Ct^ method and normalized to *β*-actin.

### 2.10. Western Blot Analysis

Liver tissues stored in liquid nitrogen were lysed with radioimmunoprecipitation assay lysis buffer mixed with protease inhibitors and phenylmethanesulfonyl fluoride. Protein concentration was calculated using the bicinchoninic acid protein assay kit (Kaiji, China). Based on the standard curve, equivalent amounts of total protein were separated in 7.5–12.5% sodium dodecyl sulfate-polyacrylamide gels and then transferred onto 0.22 *μ*m polyvinylidene fluoride membranes. Nonspecific binding sites were blocked with PBS containing 5% nonfat milk for at least 1 h at room temperature, and the membranes were incubated overnight at 4°C with the following primary antibodies and dilutions: HMGB1 (1 : 10,000), TLR4 (1 : 500), p-JNK (1 : 500), TNF-*α* (1 : 500), LC3 (1 : 1,000), Beclin 1 (1 : 1,000), Bcl-2 (1 : 1,000), Bax (1 : 1,000), caspase3 (1 : 1,000), caspase9 (1 : 1,000), and *β*-actin (1 : 1,000). The next day, membranes were washed three times with PBS containing 0.1% Tween 20 (PBST) and then incubated with horseradish peroxidase-conjugated anti-rabbit or anti-mouse secondary antibodies at 1 : 2,000 for 1 h at room temperature. After three washes with PBST, membranes were scanned with the Odyssey two-color infrared laser imaging system, and the gray values were quantified using ImageJ v1.8.0 software.

### 2.11. Statistical Analysis

Data were presented as the mean ± standard error. Differences between groups were detected by one-way analysis of variance. A *P* value of <0.05 was considered statistically significant. Statistical analyses were performed with SPSS 20.0 software (Chicago, IL, USA).

## 3. Results

### 3.1. OA Preconditioning Restores the Hepatic IR-Induced Increase in Serum Aminotransferase Levels

**Figure 1:**
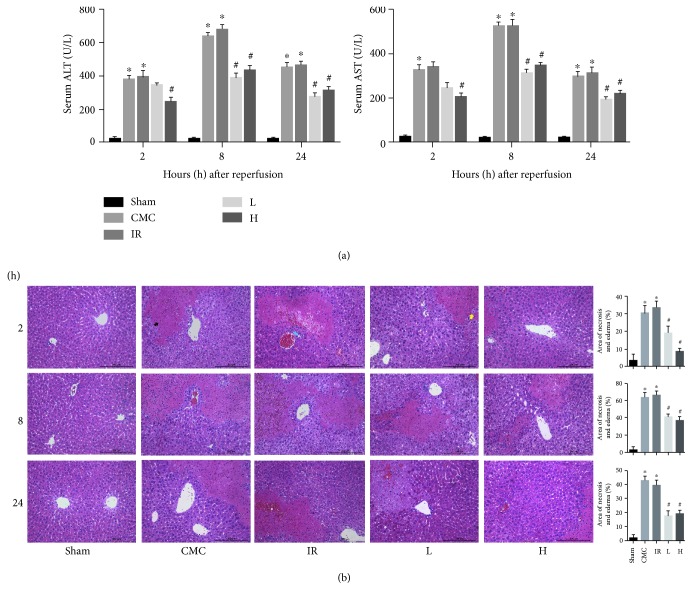
OA preconditioning alleviates hepatic IRI biochemically and histopathologically. (a) Serum ALT and AST levels at 2, 8, and 24 h after hepatic IR detected by ELISA. (b) H&E staining of the liver specimens collected at three time points. Scale bar: 200 *μ*m. The black arrow indicates the necrotic area, the yellow arrow represents edema, and the blue arrow indicates leukocyte infiltration. The areas of necrosis and edema were quantified with Image-Pro Plus 6.0 (original magnification, ×200). Data are presented as mean ± standard error (*n* = 6, ^∗^*P* < 0.05 versus sham, ^#^*P* < 0.05 versus IR).

The levels of ALT and AST were markedly higher in the IR group than in the sham group at 2, 8, and 24 h post procedure. This demonstrated the successful establishment of a segmental hepatic IR model, in which the greatest increase in aminotransferase was observed at 8 h. The elevated activities of aminotransferase at the same time points were dramatically reduced by precoditioning with 60 mg/kg OA. A 30 mg/kg dose of OA had the same effect on decreasing transaminase levels except at the 2 h time point. There was no significant difference in aminotransferase activity between the CMC group and the IR group (Figure 1(a)).

### 3.2. OA Mitigates Liver Histopathological Injury Caused by Hepatic IR

H&E staining of the liver specimens showed that hepatic lobular structures remained ordered in the sham group, whereas massive hepatic necrosis, marked neutrophil infiltration, moderate-to-severe edema, and congestion were observed in the IR and CMC groups at the three time points (Figure 1(b)). Pretreatment with OA at 30 and 60 mg/kg alleviated the histopathological changes in the IR group. No significant differences were observed between the CMC group and the IR group (Figure 1(b)). The results of pathological grading of the liver sections collected at 8 h after hepatic IR were consistent with the above results (Table 2).

### 3.3. OA Suppresses Apoptosis and Autophagy during Hepatic IR

**Figure 2:**
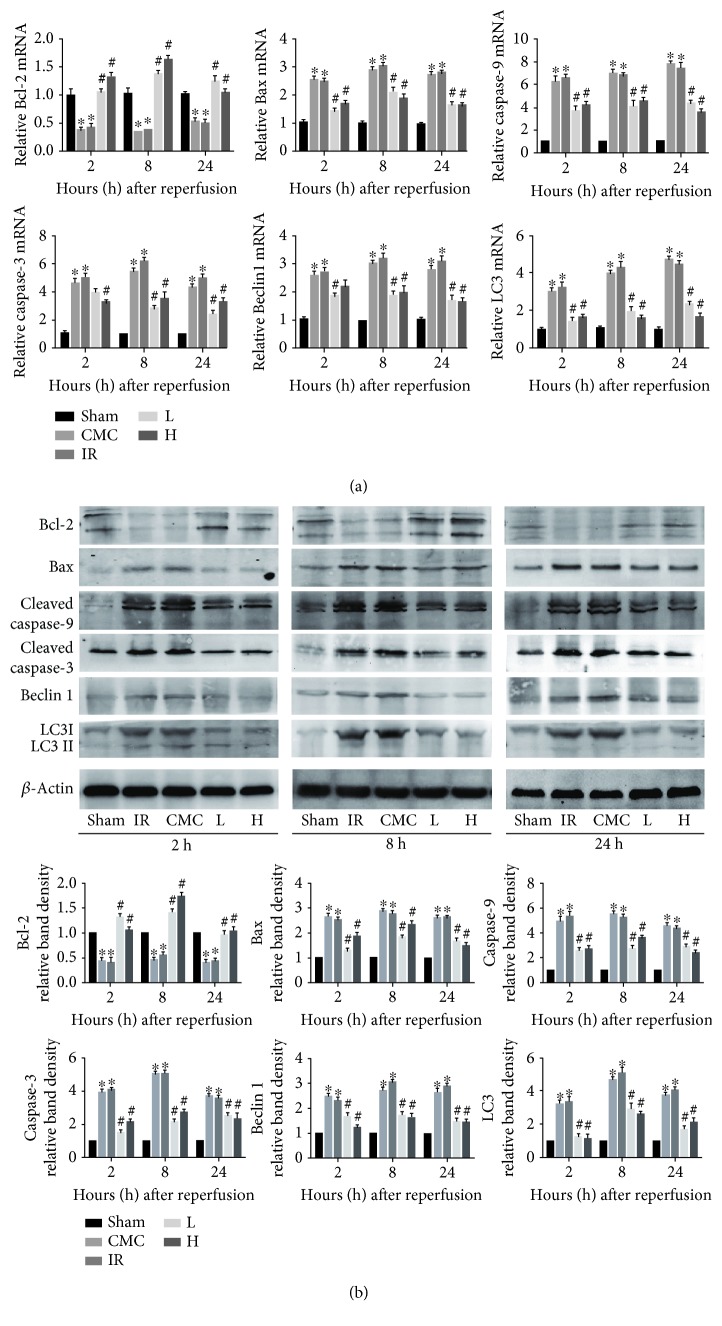
OA pretreatment attenuates the intrinsic pathways of apoptosis and autophagy in hepatic IR. (a) Liver mRNA levels of Bcl-2, Bax, caspase-3, caspase-9, Beclin 1, and LC3 at 2, 8, and 24 h after hepatic IR were quantified by real-time PCR. (b) Protein expression of Bcl-2, Bax, caspase-3, caspase-9, Beclin 1, and LC3 detected by western blotting. The western blot results were quantified with ImageJ v1.8.0 software. Data are presented as mean ± standard error (*n* = 6, ^∗^*P* < 0.05 versus sham, ^#^*P* < 0.05 versus IR).

IR induction inhibited the mRNA and protein expression of the anti-apoptotic Bcl-2 and upregulated the proapoptotic proteins caspase-3, caspase-9, and Bax. OA preconditioning upregulated Bcl-2 and downregulated Bax, caspase-9, and caspase-3 at the three time points, resulting in a decrease of the Bax/Bcl-2 ratio. Autophagy-related proteins showed a similar pattern, with high levels of expression of LC3 and Beclin 1 in the IR and CMC groups and marked downregulation of these proteins in response to OA preconditioning (Figures 2(a) and 2(b)).

### 3.4. OA Inhibits HMGB1 Production and TNF-*α*-Mediated JNK Signaling

**Figure 3:**
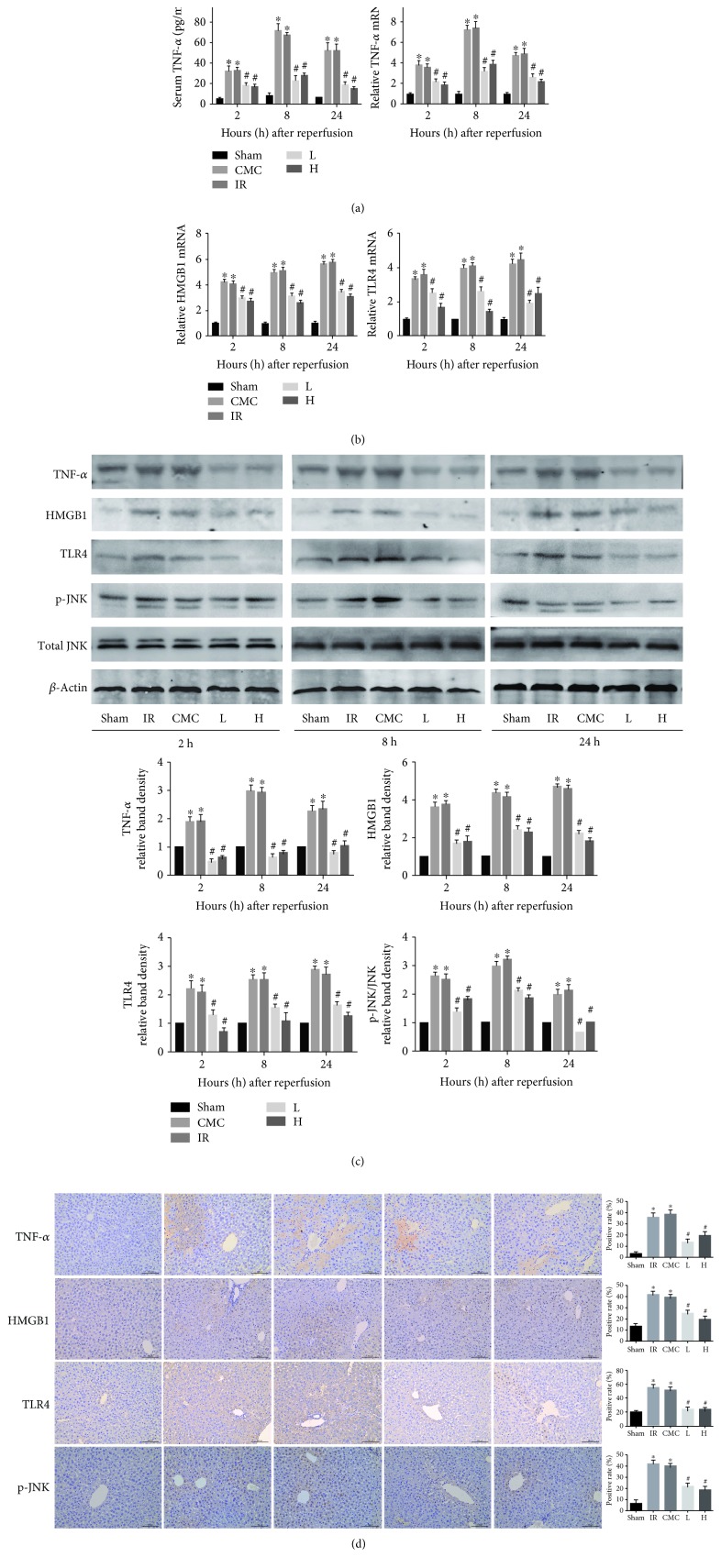
OA reduces HMGB1 production and downregulates TNF-*α*-mediated JNK signaling. (a) Serum TNF-*α* level and hepatic TNF-*α* mRNA expression detected by ELISA and real-time PCR, respectively. (b) mRNA levels of HMGB1 and TLR4 determined by real-time PCR. (c) Protein levels of TNF-*α*, HMGB1, TLR4, phospho-JNK, and total JNK at three time points evaluated by western blotting. (d) Protein levels of TNF-*α*, HMGB1, TLR4, and phospho-JNK at 8 h after hepatic IR quantified by immunohistochemistry (original magnification: 200x). Data are presented as mean ± standard error (*n* = 6, ^∗^*P* < 0.05 versus sham, ^#^*P* < 0.05 versus IR).

To explore the mechanism underlying the effects of OA, TNF-*α* levels were assessed by RT-PCR, ELISA, and western blotting, and HMGB1 and TLR4 levels were assessed by IHC. The results showed that hepatic IR increased serum TNF-*α* levels, which peaked at 8 h, whereas OA pretreatment significntly suppressed the increase in TNF-*α* (Figure 3(a)). Consistent with these results, the mRNA and protein expression of TNF-*α* were remarkably upregulated in the CMC and IR groups, whereas OA preconditioning inhibited this effect compared with hepatic IR mice (Figures 3(a), 3(c) and 3(d)). HMGB1 and TLR4 mRNA and protein levels were increased in the CMC and IR groups at the three time points, particularly at 8 and 24 h, whereas the levels were dramatically reduced by OA pretreatment. An increasing trend in JNK phosphorylation was observed in hepatic IR mice, and this effect was suppressed by pretreatment with OA suspension (Figures 3(b)–3(d)).

## 4. Discussion

Liver IRI is associated with clinically relevant processes such as liver transplantation, hepatectomy, and hemorrhagic shock [30]. In the past decades, several potential mechanisms were proposed to explain the occurrence of IRI. The aim of the current study was to explore the underlying mechanisms and suggest potential strategies for the prophylaxis and treatment of liver IRI.

OA is a natural product with anticancer, antimicrobial, and antidiabetic activities that is used as an alternative therapy for chronic liver diseases in China [10, 31]. In a previous study from our group, we demonstrated the protective effects of OA in a model of concanavalin A-induced acute hepatitis [28]. Whether OA plays a similar hepatoprotective role in a model of hepatic IRI and the underlying mechanisms needs further investigation. In the present study, we showed that OA has hepatoprotective effects against liver IRI biochemically and histopathologically. No significant differences in serum aminotransferase levels and pathological score were observed between the CMC and IR groups, indicating that the solvent, 0.5% CMC-Na, did not affect liver function. Previous studies showed that elevated HMGB1 is related to the pathophysiologic process of liver IR. The addition of HMGB1 promotes the release of TNF-*α* and TNF-*α* also facilitates the production of HMGB1 [18–21]. Liu et al. [20] and Tsung et al. [21] showed that inhibition of HMGB1 expression has hepatoprotetive effects against hepatic IR, as indicated by the alleviation of histopathological injury and decreased levels of inflammatory cytokines. These findings suggest that HMGB1 is a potential target for the treatment of hepatic IRI. OA inhibits HMGB1-mediated pathway activation in lipopolysaccharide-stimulated RAW264.7 cells [32] and human umbilical vein endothelial cells [33]. HMGB1 acts as a late mediator of the inflammatory response, activating monocytes to secrete proinflammatory cytokines, including TNF-*α* and IL-1 [34], and inducing inflammation as the ligand of TLR4 [18]. Andersson et al. provided evidence that HMGB1 increases the release of TNF *in vivo* and *in vitro* [19]. Therefore, we hypothesized that OA exerts its hepatoprotective effects by inhibiting HMGB1. The results showed that TNF-*α* and HMGB1 were significantly upregulated at the mRNA and protein levels in hepatic IR mice at three time points, supporting the proinflammatory role of HMGB1. OA pretreatment downregulated TNF-*α* and HMGB1, suggesting that OA has anti-inflammatory and HMGB1 inhibitory effects.

**Figure 4:**
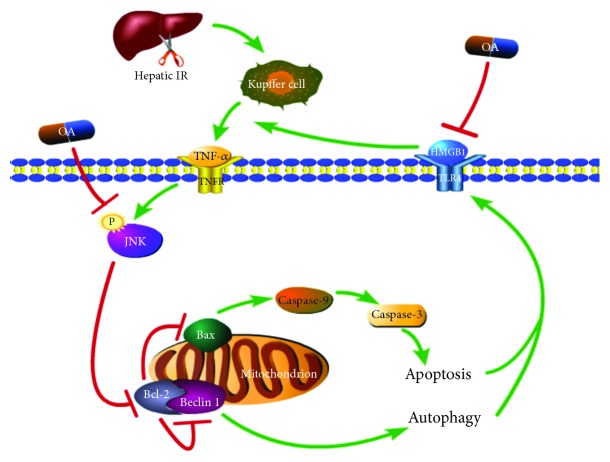
A schematic diagram of the mechanism underlying the hepatoprotective effect of OA on liver IR. In our hepatic IR model, OA inhibits TNF-*α*-mediated JNK phosphorylation, thereby decreasing hepatocellular apoptosis and autophagy by promoting Bcl-2 activity and suppressing the expression of caspase-9 induced by Bax and Beclin 1 activation. OA reduces the production of the late inflammatory mediator HMGB1, alleviating hepatic IRI.

Apoptosis and autophagy are involved in hepatic IRI. In addition, TNF-*α* activates JNK signaling, and JNK phosphorylation is closely related to the induction of cellullar autophagy and apoptosis [35–37]. Phosphorylation of JNK downregulates the antiapoptotic protein Bcl-2 and upregulates the proapoptotic protein Bax [38, 39], activating caspase-9 and caspase-3 signaling cascades leading to cellular apoptosis [40]. Bcl-2 interacts with the autophagy-related protein Beclin 1 through its BH3 (Bcl-2 homology 3) domain, thereby decreasing Beclin 1 activity, inhibiting autophagy, and limiting the conversion of LC3 (microtubule-associated protein 1 light chain 3) I to LC3 II [41, 42]. Jiang et al. reported an association between apoptosis and HMGB1 release, in which inhibition of JNK suppressed apoptosis resulting in decreased HMGB1 release in RAW 264.7 cells [43]. OA inhibits JNK phosphorylation, leading to the inhibition of downstream apoptosis and autophagy [28, 44]. This suggested that the protective effects of OA against hepatic IR are mediated by targeting JNK signaling activation and its effects on the regulation of hepatocellular apoptosis and autophagy. To clarify the contribution of the JNK pathway to the hepaptoprotective effects of OA in hepatic IR, we examined the expression levels of p-JNK, caspase-3, caspase-9, Bcl-2, Bax, Beclin 1, and LC3. The results showed that mice under IR-inducing conditions had higher levels of JNK phosphorylation in the liver, together with higher expression of Bax, Beclin 1, LC3, caspase-3, and caspase-9 at the mRNA and protein levels, and this effect was abrogated by OA administration prior to IR operation. In addition, OA upregulated the expression of the antiapoptotic protein Bcl-2, which was downregulated in the CMC and IR groups (Figure 4).

The results of the present study provide insight into the proinflammatory role of HMGB1 in the pathophysiology of hepatic IR and suggest that OA exerts its hepatoprotective effects by decreasing HMGB1 release and inhibiting apoptosis and autophagy. These findings provide a new therapeutic option for the prophylaxis and treatment of liver IRI.

## Figures and Tables

**Table 1:** Oligonucleotide primer sequences for qRT-PCR.

Gene	Forward (5′-3′)	Reverse (3′-5′)
*β*-Actin	GGCTGTATTCCCCTCCATCG	CCAGTTGGTAACAATGCCATGT
TNF-*α*	CAGGCGGTGCCTATGTCTC	CGATCACCCCGAAGTTCAGTAG
HMGB1	GGCGAGCATCCTGGCTTATC	GGCTGCTTGTCATCTGCTG
TLR4	ATGGCATGGCTTACACCACC	GAGGCCAATTTTGTCTCCACA
Bcl-2	GCTACCGTCGTCGTGACTTCGC	CCCCACCGAACTCAAAGAAGG
Bax	AGACAGGGGCCTTTTTGCTAC	AATTCGCCGGAGACACTCG
Beclin 1	ATGGAGGGGTCTAAGGCGTC	TGGGCTGTGGTAAGTAATGGA
LC3	GACCGCTGTAAGGAGGTGC	AGAAGCCGAAGGTTTCTTGGG
Caspase-3	ATGGAGAACAACAAAACCTCAGT	TTGCTCCCATGTATGGTCTTTAC
Caspase-9	TCCTGGTACATCGAGACCTTG	AAGTCCCTTTCGCAGAAACAG

**Table 2:** Pathological grading for hepatic injury 8 h after IR.

	0	1	2	3	4	Mean
Sham	3	3	0	0	0	0.50
CMC	0	0	1	1	4	3.50∗
IR	0	0	0	1	5	3.83∗
L	0	1	3	2	0	2.17^#^
H	0	3	2	1	0	1.67^#^

*n* = 6, ^∗^*P* < 0.05 versus sham, ^#^*P* < 0.05 versus IR.

## Data Availability

The data used to support the findings of this study are available from the corresponding author upon request.
